# Efficiency of ultrasound-guided placental gene therapy in a rabbit IUGR model and effects on offspring development

**DOI:** 10.3389/fvets.2025.1633597

**Published:** 2025-09-24

**Authors:** María Soledad Trigo, Antonio Gonzalez-Bulnes, Pilar García-Rebollar, Nora Formoso-Rafferty, Teresa Cejalvo, Javier García-Castro, Natalia Yeste-Vizcaíno, Alicia Barbero-Fernández

**Affiliations:** ^1^Faculty of Veterinary Medicine, Universidad Alfonso X El Sabio, Madrid, Spain; ^2^Faculty of Veterinary Sciences, Universidad Cardenal Herrera-CEU, CEU Universities, Valencia, Spain; ^3^Producción Agraria, E.T.S.I.A.A.B., Universidad Politécnica, Madrid, Spain; ^4^Agencia Española de Medicamentos y Productos Sanitarios, Madrid, Spain; ^5^Unidad de Biotecnología Celular, Instituto de Salud Carlos III, Madrid, Spain; ^6^Faculty of Veterinary Medicine, Universidad Autónoma de Barcelona, UAB, Barcelona, Spain; ^7^Vetoclock Diagnóstico y Formación S.L. Don Ramón de la Cruz, Madrid, Spain

**Keywords:** adenoviral vectors, gene therapy, IGF-1, IUGR, rabbit, VEGF

## Abstract

Placental insufficiency is a major cause of intrauterine growth restriction (IUGR), and no effective prenatal therapy is currently available. Previous studies have demonstrated that gene therapies based on overexpression of insulin-like growth factor 1 (IGF-1) and vascular endothelial growth factor (VEGF) can improve prenatal and postnatal development. Such therapies are mostly adenovirus-mediated, since adenoviral vectors are the most widely used vehicles for gene delivery due to their broad cellular tropism, ability to transduce non-dividing cells, and relative easiness of production and titration. Hence, this study evaluated the feasibility and safety of a novel minimally invasive ultrasound-guided intraplacental technique for the injection of adenoviral vectors encoding IGF-1 or VEGF in a rabbit model of IUGR, in which the condition was induced by maternal feed restriction to 50% of the average daily intake during gestation. Postnatal development was assessed through biometric measurements (body weight), metabolic profiling (lipid, carbohydrate, and protein metabolism), and histological cell counts in key organs. The results confirmed the marker expression by adenoviral vectors in all the treated fetuses and the absence of deleterious effects of the ultrasound-guided injection on the postnatal survival and growth of the offspring. The use of the technique for inducing both IGF-1 and VEGF over-expression by administration of adenoviral vectors in an IUGR model showed, in the case of VEGF, positive effects on the developmental and metabolic traits, with especial significance in the pulmonary and intestinal systems, of IUGR offspring. Hence, after further research, the proposed therapy may offer potential benefits in pregnancies with placental insufficiency and IUGR conditions.

## 1 Introduction

Intrauterine growth restriction (IUGR), the inability of fetuses to fully develop their potential growth, currently affects up to 10% of human pregnancies (1, 2). IUGR has been associated with acute medical complications, such as perinatal asphyxia by delayed pulmonary maturation and reduced surfactant production, hypothermia, prematurity, and death (3). Furthermore, studies of human populations and experimental animals exposed to suboptimal conditions during pregnancy have demonstrated a relationship between low birth weight and an increased risk of developing metabolic disorders (altered adipose tissue distribution, insulin resistance, diabetes, dyslipidemia, and metabolic syndrome), cardiovascular alterations (structural and functional changes in the heart and vasculature leading to increased lifetime risk of hypertension, ischemic heart disease, and stroke), and other failures in different organs like the lungs, kidneys, or intestine (4).

IUGR onset is mostly related to an inadequate intrauterine availability of nutrients and/or oxygen. IUGR is traditionally found in low-income countries and related to maternal malnutrition (e.g., socioeconomic disadvantage, food shortages, or inadequate diets) or maternal hypobaric hypoxia (e.g., pregnant women living in or visiting high altitudes). However, currently, there is a strong increase of IUGR incidence in developed countries, which is linked to placental factors (abnormal placental development and/or function and shortage of transfer of nutrients and oxygen to the fetus (5)). Such a condition, named placental insufficiency, represents one of the leading causes of IUGR cases nowadays (6).

The most worrying aspect is that placental insufficiency is expected to significantly increase in the near future since it is highly related to different pathological and behavioral factors (i.e., preeclampsia, obesity, diabetes, stress, malnutrition, sedentary habits, delays in childbearing age, pollution, and alcohol and tobacco consumption). Another worrying factor is that there are no actions with proven validity to prevent or improve placental insufficiency, so current treatments are just based on changes in lifestyle and diet. Hence, there is a strong need for preventive and therapeutic strategies. Such treatments should be based on either favoring the development of the placental/fetal tissues or favoring the processes of placental angiogenesis and vasodilation.

Development of fetal/placental tissues and, specifically, fetal growth and muscle development is activated, among other factors, by insulin and Insulin Growth Factor 1 (IGF-1) (7). IGF-1 signaling is essential during prenatal development because, in addition to Growth Hormone (GH), it regulates metabolic processes including protein synthesis and tissue development of the fetoplacental unit (8, 9). Moreover, links between deficiencies in the IGF-1 pathway and prenatal and postnatal consequences of IUGR have been clearly established (10, 11). Placental insufficiency is also related to vascular disorders of the organ (12).

Current evidence highlights the links between IUGR and deficiencies in proangiogenic factors, mainly in the vascular endothelial growth factor (VEGF) and the nitric oxide (NO) route. VEGF is considered a critical regulator of lipid metabolism, and suppression of the glycoprotein VEGF-D signaling leads to decreased expression of genes associated with triglyceride and cholesterol synthesis (13, 14). VEGF also promotes angiogenesis, vasodilatation, and vascular permeability (15) through production of NO and prostaglandin I2 (PGI2) (16). Thus, it has been described that the use of low-dose aspirin would be useful to counteract IUGR in case of placental insufficiency (17) because acetylsalicylic acid increases the production of PGI2. However, of the pros of routine administration of aspirin remain controversial, with many cons noted too. NO is the main vasodilator agent in the placenta, facilitating fetal homeostasis, development, and growth (18). The amino acid L-arginine, a substrate for the enzyme NO synthase (NOS), plays a key role in the production of NO and, therefore, maternal administration of arginine has been widely studied (19).

In any case, these therapies are based on oral administration of high doses of the substances to the mother to obtain high concentrations at systemic levels and, therefore, therapeutic concentrations at the fetoplacental level, which, moreover, may be confronted by inadequate placental transfer. Hence, there is a strong trend for research on other strategies, based on fetal-focused treatments, for administering treatments either directly to the fetus or through infusion of umbilical cord, amniotic sac, or placenta. Obviously, due to the high risk of fetal injury in case of direct administration or fluid/blood loss in case of administration in the umbilical cord, placenta, or amniotic sac (20), such research has to be performed in preclinical animal models.

Currently, studies on fetal-focused strategies are mostly based on the administration of gene therapies, specifically for inducing adenovirus-mediated (Ad) overexpression of growth factors, mainly IGF-1 and VEGF, in view of their importance as previously described. Adenoviral administration of IGF-1 has been found to increase pre- and post-natal offspring development and prevent liver, musculoskeletal, and cardiovascular dysfunctions associated with IUGR in mice, rabbits, and sheep (21, 22), while administration of VEGF has been found to increase uterine flow and improve fetal growth in sheep models (23).

However, experimental data on fetal-focused therapies have been only obtained by using highly invasive methods for inoculation of the Ad (exteriorization of the genital tract by laparotomy or catheterization), so the technique cannot be extrapolated to clinical practice yet. We hypothesize that adenoviral administration of IGF-1 and VEGF may be performed by ultrasound-guided injection using an Adenoviral vector 5, reaching similar effects on IUGR development to those previously described (21–23). Hence, using a rabbit model for pregnancy and IUGR, our main aims were to assess (a) the feasibility and efficiency of an alternative minimally invasive technique, the ultrasound-guided injection, for administering an Ad expressing a reporter gene (Ad5-βgal) at either placenta or amniotic sac and (b) possible effects of IGF-I or VEGF overexpression, induced with a single Ad administration with such a technique, on postnatal growth, organ architecture, and the metabolism of the offspring.

## 2 Materials and methods

This study reports the results of three successive trials, in which a total of 176 rabbit fetuses from 26 multiparous New Zealand x California crossbreed females were involved. The first trial determined the safety and possible long-term effects of the ultrasound-guided fetal puncture by assessing pre and postnatal development of the offspring. The second trial evaluated the efficiency of the ultrasound-guided administration of an adenoviral vector expressing a reporter gene with βGalactosidase (Ad5-βgal), which is widely employed as a marker in a broad range of biological assays due to its ability to generate a chromogenic compound in the presence of an appropriate substrate (24, 25). The third trial assessed possible changes in the postnatal development of IUGR fetuses after adenoviral delivery of growth factors.

All the experimental procedures were carried out on the experimental farm of the Polytechnic University of Madrid (UPM, Madrid, Spain), according to the European Union Directive and the Spanish Policy for Animal Protection RD53/2013, which establishes the management of animals used in experimentation and other scientific purposes, including teaching. The Committee of Ethics in Animal Research of the Polytechnic University of Madrid (UPM, Madrid, Spain) approved the experimental procedure (*Safety of intraplacental injection during the prenatal period in rabbits, with authorization date of December 22, 2020*). All the does were fed *ad libitum* with a commercial diet containing 16% crude protein, 37% crude fiber, and 3.7% fat with a gross energy content of 2,400 kcal/kg (Nanta, Madrid, Spain) in individual cages under controlled conditions of temperature (18–24 °C), light (14 h with light and 10 h with darkness), and relative humidity (60–75% RH) and free access to drink water. The average gestational length in New Zealand x California crossbreed females is 31 days and the day of the artificial insemination was considered as Day 0 of pregnancy. Ultrasound-guided punctures and therapies were performed on Day 26 of pregnancy, just before the period of overt growth arrest in IUGR offspring, which becomes apparent at around 85–90% of the total length of pregnancy (26).

### 2.1 First trial: safety and long-term effects of ultrasound-guided puncture

The first trial involved a total of 59 offspring from 11 clinically healthy 12 month-old females, distributed in four experimental groups. Two potential therapeutic groups were used to assess the safety of the administration of saline solution either in the placenta (Group PLA, *n* = 14) or in the amniotic fluid (Group AMN, *n* = 15); two control groups, without ultrasound-guided fetal puncture, were considered to determine possible effects of the handling procedure, either with or without female sedation (Group CONSED, *n* = 16, and Group CON, *n* = 14, respectively).

On Day 26 of pregnancy (considering Day 0 as day of artificial insemination), does from Groups PLA, AMN, and CONSED were sedated with i.m. midazolam (0.8 mg/kg; Midazolam 5 mg/mL, Normon Laboratories, Madrid, Spain) and butorphanol (0.4 mg/kg; Torbugesic Vet 10 mg/mL, Zoetis, Madrid, Spain). After that, the females were maintained in dorsal recumbence and, after shaving the abdominal area, pregnancy was confirmed with an ultrasound machine (Omega, Esaote, Barcelona, Spain) fitted to a linear probe (4–11 MHz); mean body diameters were assessed for determining adequate fetal development for gestational age. At once, 300 μL of saline solution (FisioVet, Braun, Madrid, Spain) was administered with a 23 G needle (0.6 × 60 mm Sterican, Braun, Madrid, Spain) to all fetuses in Groups PLA and AMN. Adequacy of administration was checked by visualization of bubbles in the injection area as well as by identifying the hyperechoic dotting corresponding to the needle puncture.

In all the does, natural delivery was allowed, and all live-born kits were individually assessed and weighed at birth. After weaning at 4 weeks of age, the offspring were housed in individual flat-deck cages under standard husbandry conditions for rabbits. During the first 13 weeks of life, animal developmental (body weight) and health status (survival rate) were recorded every 2 weeks.

### 2.2 Second trial: efficiency of ultrasound-guided administration of the adenoviral vector expressing a reporter gene

The second trial involved a total of 17 rabbit fetuses from two clinically healthy 12 month-old females. On Day 26 of pregnancy, both does were sedated and handled as previously described for the first trial. The adenoviral vector expressing *β*-galactosidase (Ad5-βgal at a dose of 7×10^7^ plaque-forming units (PFU)/ml, Vector Biolabs, Malvern, United States) was carried out with an injection volume of 300 μL and administered with a 23G needle at the placenta of all the fetuses, excepting the most proximal to the ovaries, which were used as controls for assessing possible negative effects of the contrast agent or the adenoviral vector.

Three days after administration of the adenoviruses, both does were sacrificed by sedation and cervical dislocation, in agreement with the euthanasia method for rabbits (Annex III of RD 53/2013). Immediately, the complete uterus was exteriorized and the fetuses were recovered one by one at their implantation sites. At once, two samples, of around 500 mm^3^ each, of lung, liver, kidney, intestine, heart, and placenta were obtained in all the fetuses. Each sample was macroscopically assessed for any possible tissue damage and immediately kept in phosphate buffer saline (PBS; Phosphate Buffer Saline pH 7.0, Reagecon, Lismacleane, Ireland) at −80 °C for 24 h and afterwards were fixed in 0.5% glutaraldehyde with PBS and Mg^2+^ ion on dry-ice for 2 h (26). After glutaraldehyde fixation, PBS washes were performed to remove the residual fixator, and the samples were incubated with an X-gal reaction buffer for 3 h at 37 °C. The X-gal reaction buffer contained 30 mM of potassium ferrocyanide, 30 mM of potassium ferricyanide, 2 mM of MgCl2, 0.02% Nonidet P-40, which is a non-ionic and non-denaturing detergent, and 0.01% sodium deoxycholate in PBS. After incubation with X-gal, the tissue was rinsed five times with PBS and, after that, it was ensured that the solution was no longer yellow.

The microscopic study was performed after embedding in a sucrose solution (Sucrose Crystalline S5-3, Thermo Fisher Scientific, Madrid, Spain) and subsequently cryopreserved to prevent ice crystal formation in tissues when water freezes and expands. After that, samples were cut in 2 μm sections and mounted on slides. Microscopic evaluation was performed using the digital camera of the microscope (BA210 LED with 1080INT; Motic, Barcelona, Spain), assessing firstly any possible tissue damage; after that, the expression level of the Ad was quantified as 1 (mild or focal expression), 2 (moderate and/or multifocal expression), or 3 (intense and diffuse expression). Three sections per sample were evaluated and the values were averaged for each tissue in each animal.

### 2.3 Third trial: effects of the growth factor overexpression using ad therapy on postnatal development of IUGR fetuses

The third trial involved a total of 100 rabbit fetuses from 13 clinically healthy 12 month-old females. On Day 9 of pregnancy, does were randomly distributed in two groups for later assessment of the developmental patterns of IUGR fetuses. The first group (Group Control or CON, 42 fetuses from five females) was fed *ad libitum* throughout pregnancy. The IUGR condition was induced through maternal dietary restriction in the second group (Group IUGR, *n* = 58 fetuses from eight females) using a IUGR model previously developed at our laboratory (27). Summarizing, from Day 9 of pregnancy to the day of delivery, the does in Group IUGR were restricted to 50% of their average daily food intake. Such daily intake was calculated to be 187.0 ± 11.0 g/day. Afterwards, Group IUGR was randomly divided into three subsets based on the therapy received at the placenta. A first subset received no treatment (Group IUGR-CON, *n* = 28 fetuses from three females), while two subsets were treated with ultrasound-guided intraplacental gene therapy at Day 26 of pregnancy, as described for the second trial. Vascular Endothelial Growth Factor (VEGF; Group IUGR-VEGF, *n* = 19 fetuses from three females) was overexpressed by Ad-VEGF at a dose of 1×10^10^ PFU/ml (Vector Biolabs, Malvern, United States); Insulin-like Growth Factor 1 (IGF-1; Group IUGR-IGF, *n* = 11 fetuses from two females) was overexpressed by Ad-IGF1 at a dose of 5×10^10^ PFU/ml (Vector Biolabs, Malvern, United States). In all these does, as described for the first trial, natural delivery was allowed and, afterwards, *ad libitum* access to drinking water and feed was allowed during lactation.

All kits born alive were individually identified and, after weaning at 4 weeks of age, were individually housed, throughout the complete study, under identical environmental conditions and feeding patterns to their mothers after delivery (i.e., *ad libitum* access to drinking water and to the feed diet was allowed). Health status and developmental and metabolic traits were monitored. Body weight was evaluated every 2 weeks from Day 3 to Day 95 after birth in all groups and at Day 135 in the IUGR groups. Plasma concentrations of biomarkers for lipids (non-esterified fatty acids [NEFA], total cholesterol, high-density lipoprotein cholesterol [HDL-c], low-density lipoprotein cholesterol [LDL-c], and triglycerides), carbohydrates (glucose and fructosamine), and proteins (lactate and urea) were determined only in the IUGR Groups, due to animal welfare limitations. At 3, 30, 95, and 135 days-old, blood samples were drawn at the lateral saphenous vein with heparin-treated 1 mL tubes (Aquisel; La Bouvet, Madrid, Spain), centrifuged at 700 rpm for 15 min at 4 °C, and immediately stored at −80 °C until analysis.

At Day 135, all kits were euthanized with an i.v. dose of pentobarbital (30 mg/kg; Dolethal, Madrid, Spain) and tissue samples were collected for histomorphometric evaluation of the lung, liver, kidney, and intestine in the offspring of the IUGR groups. Sections of 500mm^3^ were obtained at the duodenum, left caudal lobe of the lung, right lobe of the liver, and medullary area of the left kidney and immediately fixed in 4% paraformaldehyde and 70% ethanol after for 24 h. Afterwards, samples were embedded in paraffin, sectioned at 4 μm thickness, and stained with hematoxylin–eosin following routine laboratory procedures. The histological evaluation of the organs was performed by a trained pathologist blinded to the experimental procedure and included the measurement of intestine in the intestinal crypts (the measurement of the distance between the mouth to the base of the crypt and the number of crypts/mm^2^) and the intestinal villi (the distance between the apex to the base of the intestinal villus and number of villi/mm), the measurement of the lung (number of alveoli and percentage of alveolar space), and measurement of the hepatocytes at the liver and the nephrons at the kidney.

### 2.4 Statistical analysis

All data analysis was performed with the SPSS 28.0 program for Windows. The analysis assessed the effects of the treatments on the survival rate, developmental traits (changes in weight over time), metabolic and welfare status, and histological findings of the kits, which were considered the experimental unit; the model also took doe and sex into consideration. For a comparison of single-point data, parametric analysis of variance (ANOVA) or the non-parametric Kruskal–Wallis test were performed, according to the data distribution; Bonferroni *post hoc* was used to determine differences among groups. Changes over time were assessed via ANOVA for repeated measurements (split-plot ANOVA), considering time and sex as fixed effects, after using a Shapiro–Wilk test for verification of normal distribution. Statistical treatment of results expressed as percentages was performed after arcsine transformation of the values for each individual percentage. All results were expressed in the manuscript as mean ± S. D. and statistical significance was accepted from *p* < 0.05, whereas a trend was considered when 0.1 > *p* > 0.05.

## 3 Results

### 3.1 First trial: safety and long-term effects of ultrasound-guided fetal puncture

**Figure 1:**
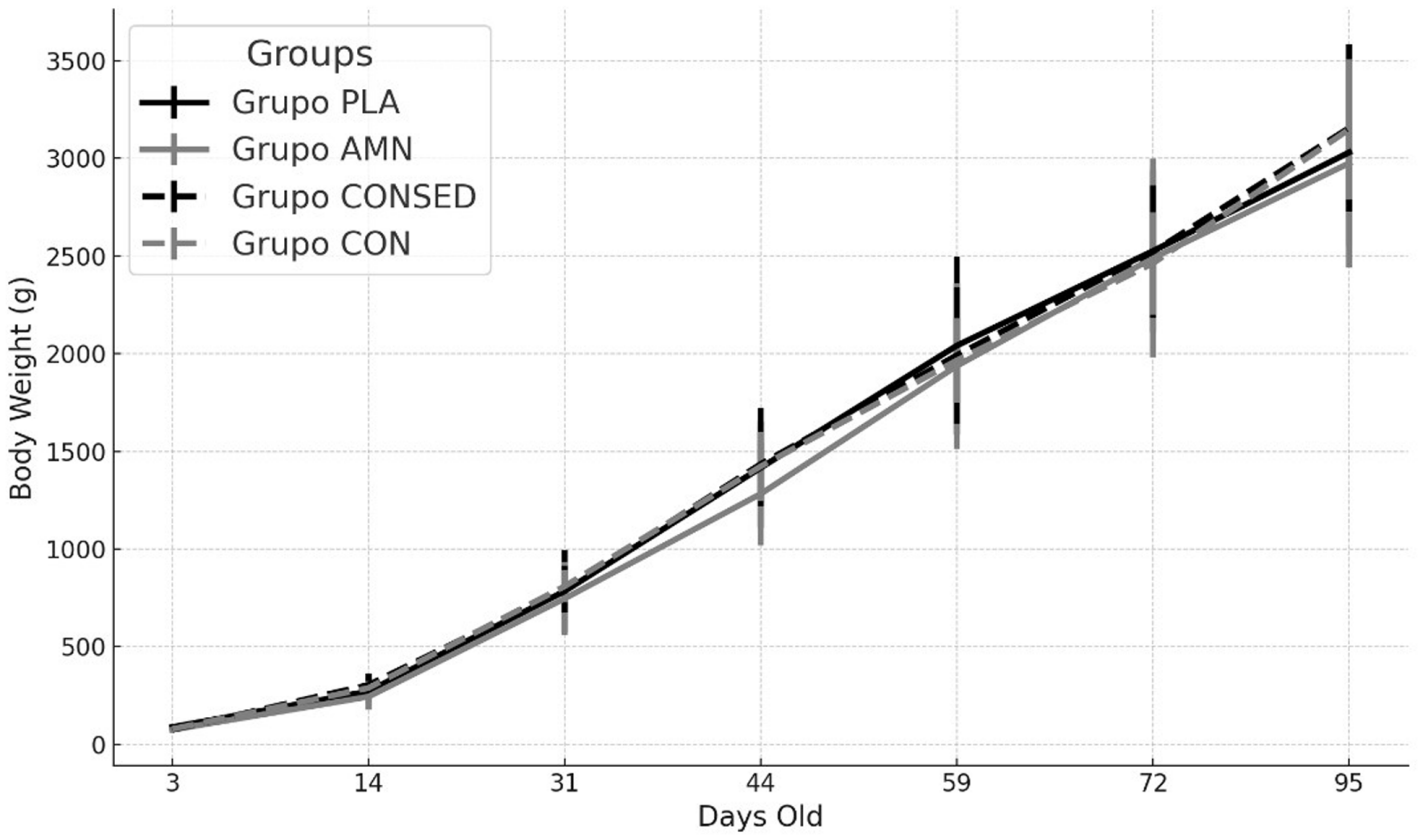
Changes over time in mean body weight (g ± S. D.) of offspring born after puncture of the placenta (Group PLA) or the amniotic fluid (Group AMN) at Day 26 of pregnancy or in controls without puncture and with or without sedation (Groups CONSED and CON, respectively).

There were no significant effects of the sedation or the fetal puncture, either at the placenta or the amniotic fluid, on sex distribution (around 52% of female vs. 48% of male kits for all the groups), offspring mortality (around 95% for all the groups), or the developmental trajectory of the offspring, which increased in weight rapidly and similarly throughout the study (*p* < 0.001), again without significant differences in body-weight changes among groups (*p* = 0.644) (Figure 1).

### 3.2 Second trial: efficiency of ultrasound-guided administration of the adenoviral vector

**Figure 2:**
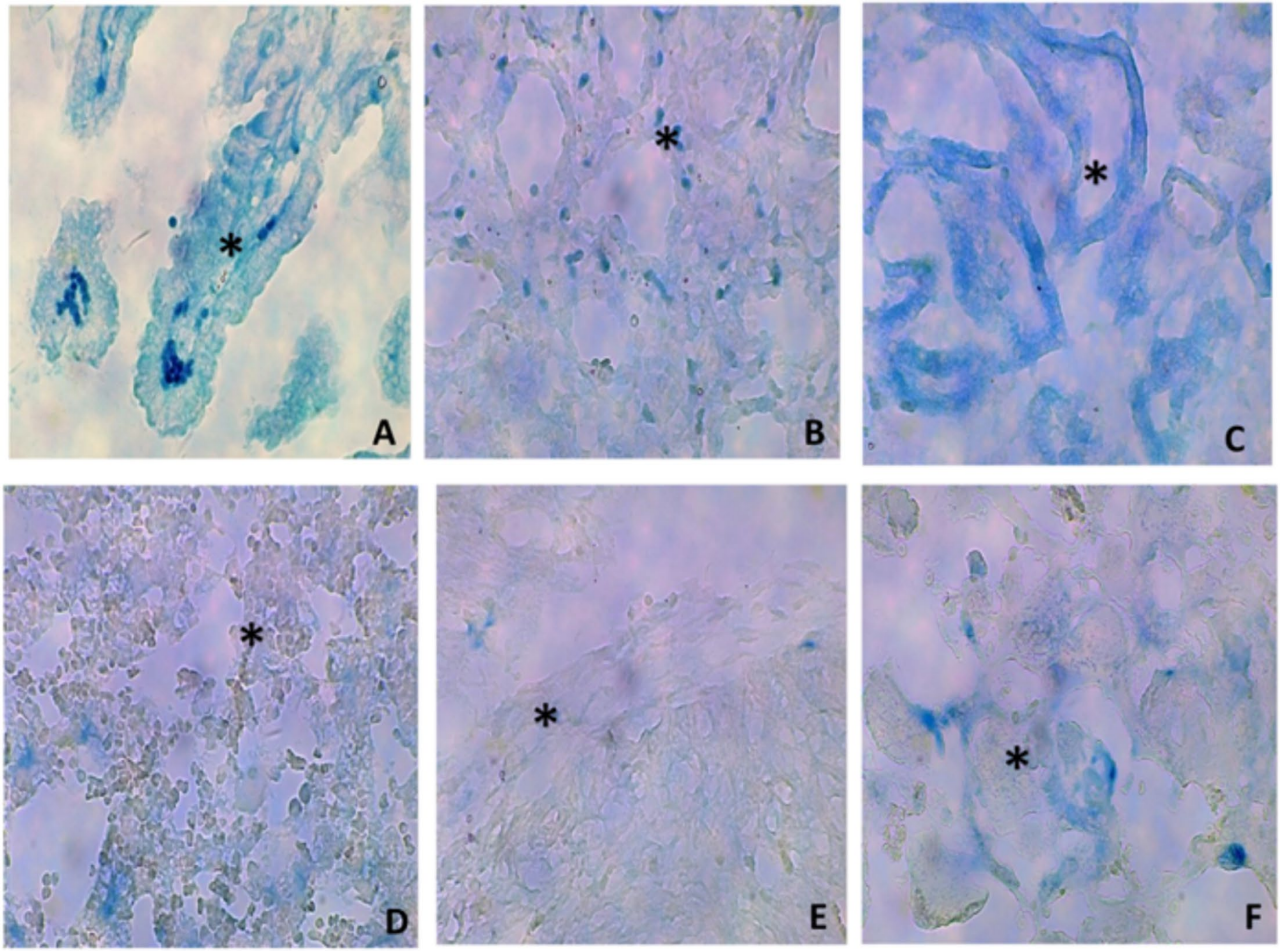
Histological images (40x) of representative micrographs showing positive *β*-galactosidase expression (asterisks) in different organs [**(A)** intestine; **(B)** lung; **(C)** kidney; **(D)** liver; **(E)** heart; **(F)** placenta] of fetuses treated with an Ad-βgal on day 26 of pregnancy.

The assessment of the contrast agent at different organs confirmed the successful administration of Ad-*β*gal to all the fetuses. Three days afterwards, macroscopic and microscopic assessment of the organs showed no tissue damage and positive expression of β-galactosidase in all the samples (Figure 2), with the highest expression in the intestine and lung (Table 1).

**Table 1:** Positive β-galactosidase expression in different organs (intestine, lung, kidney, liver, heart, and placenta) of fetuses treated with an Ad-βgal on day 26 of pregnancy.

	Intestine	Lung	Kidney	Liver	Heart	Placenta
*N*	17	17	17	17	17	17
Mean	2.53	2.41	2.12	2	1.65	1.18
S.E.M.	0.174	0.150	0.118	0.086	0.147	0.095
Median	3	2	2	2	2	1
S.D.	0.717	0.618	0.485	0.354	0.606	0.393
Minimum	1	1	1	1	1	1
Maximum	3	3	3	3	3	2
25 Percentile	3	2	2	2	2	1
50 Percentile	3	2	2	2	2	1
75 Percentile	3	2	2	2	2	1

### 3.3 Third trial: effects of the growth factor overexpression using adenoviral therapy on postnatal development of IUGR fetuses

**Figure 3:**
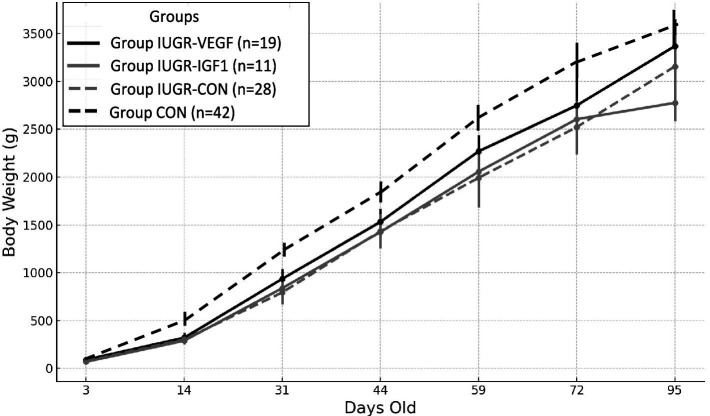
Changes over time in mean body weight (g ± S.E.M.) from 3- to 95 days-old in IUGR offspring born with Ad-IGF-1 (Group IUGR-IGF1) and Ad-VEGF (Group IUGR-VEGF) or without (Group IUGR-CON) therapies or *ad libitum* control (Group CON).

Feed restriction was successful for inducing the IUGR condition, since it significantly reduced the offspring birth weight in Group IUGR-CON when compared with a*d libitum* controls (77.22 ± 1.12 g vs. 88.44 ± 1.6 g in the Group CON; *p* = 0.022). After that, Group IUGR-CON offspring remained significantly lighter than Group CON offspring during the period of postnatal assessment (Figure 3; *p* < 0.005). There were no significant differences in sex distribution among the different groups (around 55% female vs. 45% male kits) and sex did not have a significant effect on differences in developmental trajectories among groups.

Assessment of the Ad therapies showed that the birth weight of the offspring in Group IUGR-IGF (76.01 ± 1.39 g) was similar to individuals in Group IUGR and, therefore, lower than offspring in Group CON (*p* = 0.019); such differences were maintained during postnatal development (*p* < 0.005). At 95 days-old, body weight in Group IUGR-IGF was lower than in Group IUGR (*p* = 0.025), but this difference was lost on Day 135 (3849.0 ± 353.6 g in Group IUGR-IGF vs. 3755.8 ± 186.4 g in Group IUGR-CON; *p* = 0.973).

Conversely, the birth weight of the offspring in Group IUGR-VEGF (86.75 ± 1.32 g) was similar to Group CON and, although differences did not reach statistical significance, were higher than IUGR-CON and IUGR-IGF offspring. Afterwards, offspring in Groups IUGR-VEGF showed the highest postnatal increases in body weight (*p* = 0.004); as a consequence, body weight from 31 days-old to the end of the study was only numerically lower than in Group CON and significantly higher than in Groups IUGR-CON and IUGR-IGF (*p* < 0.005).

**Figure 4:**
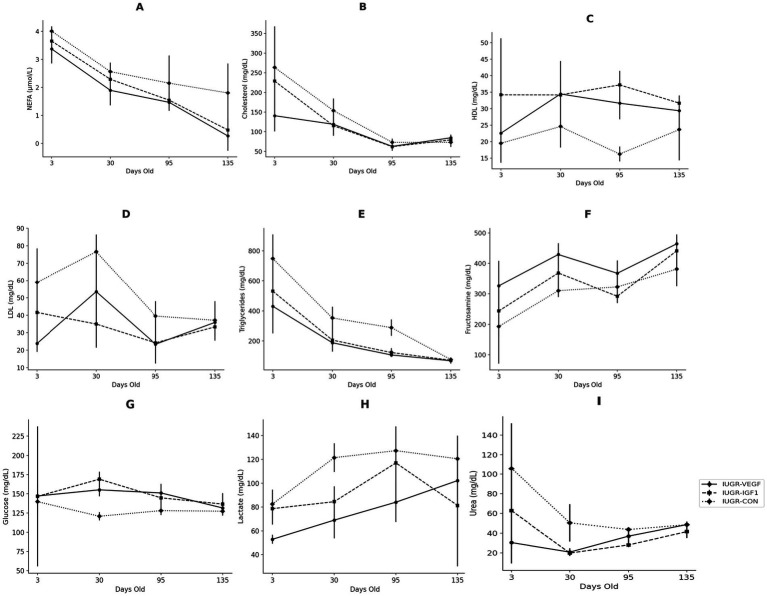
Changes over time in the biochemical parameters of IUGR offspring born with Ad-IGF-1 (Group IUGR-IGF1) and Ad-VEGF (Group IUGR-VEGF) or without (Group IUGR-CON) therapies [**(A)** NEFA, **(B)** Total cholesterol, **(C)** HDL-c, **(D)** LDL-c, **(E)** Triglycerides, **(F)** Fructosamine, **(G)** Glucose, **(H)** Lactate, **(I)** Urea].

The assessment of the metabolic indexes during postnatal development showed significant effects of the intraplacental therapies on the biochemical parameters of the offspring (Figure 4). The evolution of the parameters from 3- to 135 days-old was increasing in nearly all parameters, although NEFA, cholesterol, and triglycerides decreased and glucose remained stable. Specifically, NEFA levels decreased significantly from day 3 to 30 (*p* < 0.001), numerically from day 30 to 95 (*p* = 0.052), and significantly again from day 95 to 135 (*p* < 0.001). Group IUGR-VEGF showed significantly lower NEFA concentrations (1.75 ± 0.04 vs. 2.63 ± 0.01 μmol/L in the IUGR-VEGF and IUGR-CON groups, respectively; *p* < 0.001). Cholesterol concentrations also showed significant differences among groups throughout the study (*p* < 0.001). High-Density Lipoprotein (HDL-c), was significantly higher (*p* < 0.001) in Group IUGR-IGF1 than in Group IUGR-CON and numerically higher (*p* = 0.071) in Group IUGR-VEGF than in Group IUGR-CON (34.32 ± 1.95, 29.51 ± 1.94 and 20.99 ± 1.94 mg/dL, respectively). Conversely, low-density lipoprotein (LDL-c) was lower in both Group IUGR-IGF1 and IUGR-VEGF than in Group IUGR-CON (33.50 ± 3.95 and 34.13 ± 0.27 vs. 53.03 ± 3.48.42 mg/dL, respectively; *p* < 0.01). Triglycerides in Group IUGR-VEGF were lower than in the IUGR-CON Group (198.48 ± 38.05 vs. 366.52 ± 80.47 mg/dL, respectively; *p* < 0.05). Glucose concentrations showed a higher increase over time in Group IUGR-VEGF (*p* < 0.001). Fructosamine values showed a sigmoidal trajectory in all groups, rising and falling uniformly, except for in Group IUGR-CON, which showed an upward trend on Day 95 and then returned to behaving like the others (*p* < 0.05). On day 30, Group IUGR-IGF1 showed lower fructosamine concentrations than Group IUGR-CON (336.50 ± 16.61 vs. 301.91 ± 26.61 mg/dL; *p* < 0.001). For lactate, Groups IUGR-VEGF and IUGR-IGF1 showed lower values than Group IUGR-CON throughout the study (77.01 ± 4.31 and 90.36 ± 3.14.090 vs. 112.94 ± 4.15 mg/dL, respectively; *p* < 0.001). Rabbits in Group IUGR-VEGF showed lower levels of urea than those in the IUGR-CON group (41.89 ± 5.60 vs. 62.13 ± 4.70 mg/dL, respectively; *p* < 0.05), and, although not statistically significant (*p* = 0.079), the IUGR-IGF1 Group was 29.43 mg/dL lower than the IUGR-CON control group. In summary, compared to IUGR-CON, IUGR-IGF offspring showed lower plasma concentrations of LDL-c (*p* = 0.007), lactate (*p* < 0.001), and urea (*p* = 0.079) and higher concentrations of fructosamine (*p* < 0.001) and HDL-c (*p* < 0.001) throughout the study period. Again, the greatest differences with IUGR-CON were detected in the IUGR-VEGF offspring, with higher concentrations of glucose (*p* < 0.001) and HDL-c (*p* = 0.071) and lower levels of triglycerides (*p* = 0.015), LDL (*p* = 0.007), NEFA (*p* < 0.001), lactate (*p* < 0.001), and urea (*p* = 0.013) (Table 2).

**Table 2:** Mean values (mean ± S.D.) throughout the period of study (from days 3 to 135) of the main biomarkers for lipid, carbohydrate, and protein metabolism in offspring born after Ad-VEGF (Group IUGR-VEGF), Ad-IGF1 (Group IUGR-IGF1), or no (Group IUGR-CON) therapy.

	IUGR-VEGF	IUGR-IGF1	IUGR-CON	*p*
NEFA (μmol/L)	1.828 ± 1.556	2.241 ± 1.464	2.397 ± 1.416	*p* < 0.001
Cholesterol (mg/dL)	103.581 ± 63.479	122.167 ± 75.365	118.415 ± 62.690	*p* < 0.001
HDL-c (mg/dL)	31.346 ± 7.934	35.681 ± 3.817	20.650 ± 7.454	*p* < 0.001
LDL-c (mg/dL)	43.836 ± 38.485	32.135 ± 15.492	45.084 ± 18.360	*p* = 0.007
Triglycerides (mg/dL)	209.805 ± 183.750	341.440 ± 371.956	417.752 ± 374.428	*p* = 0.015
Fructosamine (mg/dL)	373.542 ± 91.998	353.007 ± 80.234	297.255 ± 75.678	*p* < 0.001
Glucose (mg/dL)	143.280 ± 8.520	159.652 ± 36.258	129.396 ± 11.236	*p* < 0.001
Lactate (mg/dL)	69.252 ± 13.530	106.739 ± 40.338	115.490 ± 17.339	*p* < 0.001
Urea (mg/dL)	32.183 ± 9.969	30.671 ± 12.675	57.179 ± 13.092	*p* = 0.079

**Figure 5:**
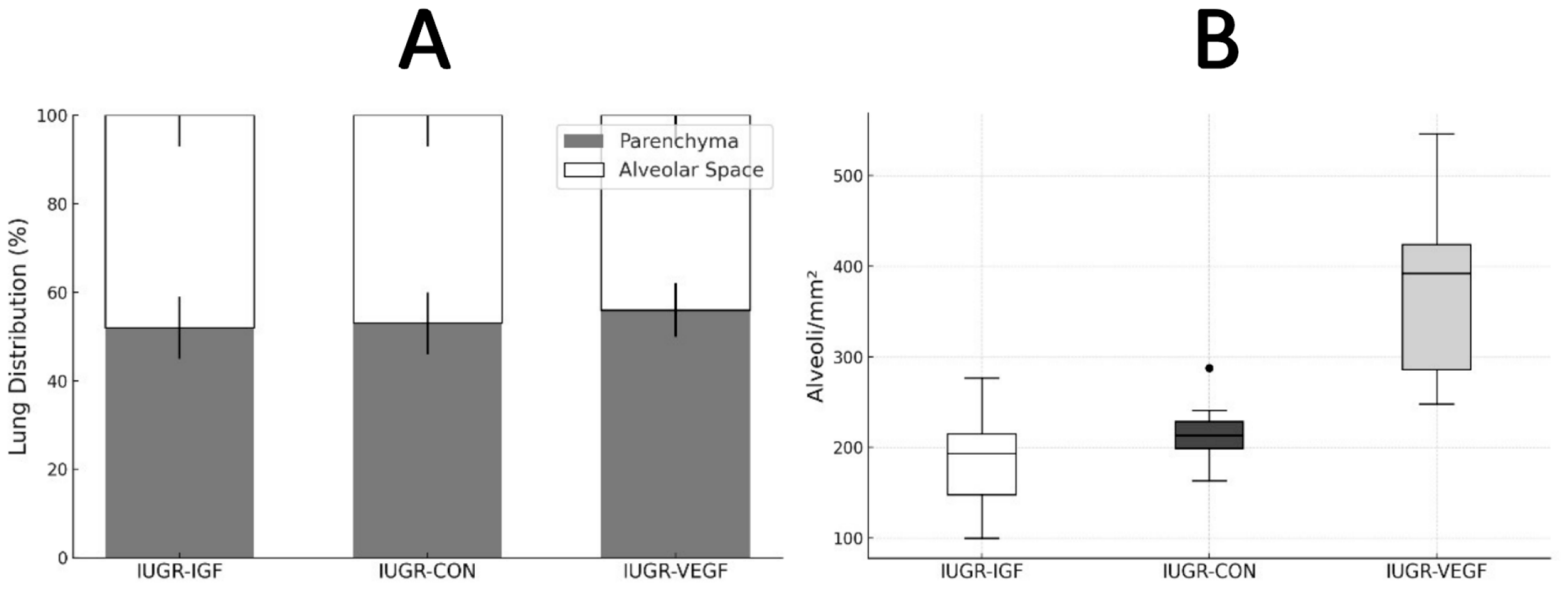
Histomorphometric analysis of the lung at the end of the study in rabbits after receiving IUGR condition (IUGR-CON) and Ad-VEGF (IUGR-VEGF) or Ad-IGF1 (IUGR-IGF1). **(A)** Lung distribution (%). **(B)** Number of alveoli/mm^2^ (unit/mm^2^).

**Figure 6:**
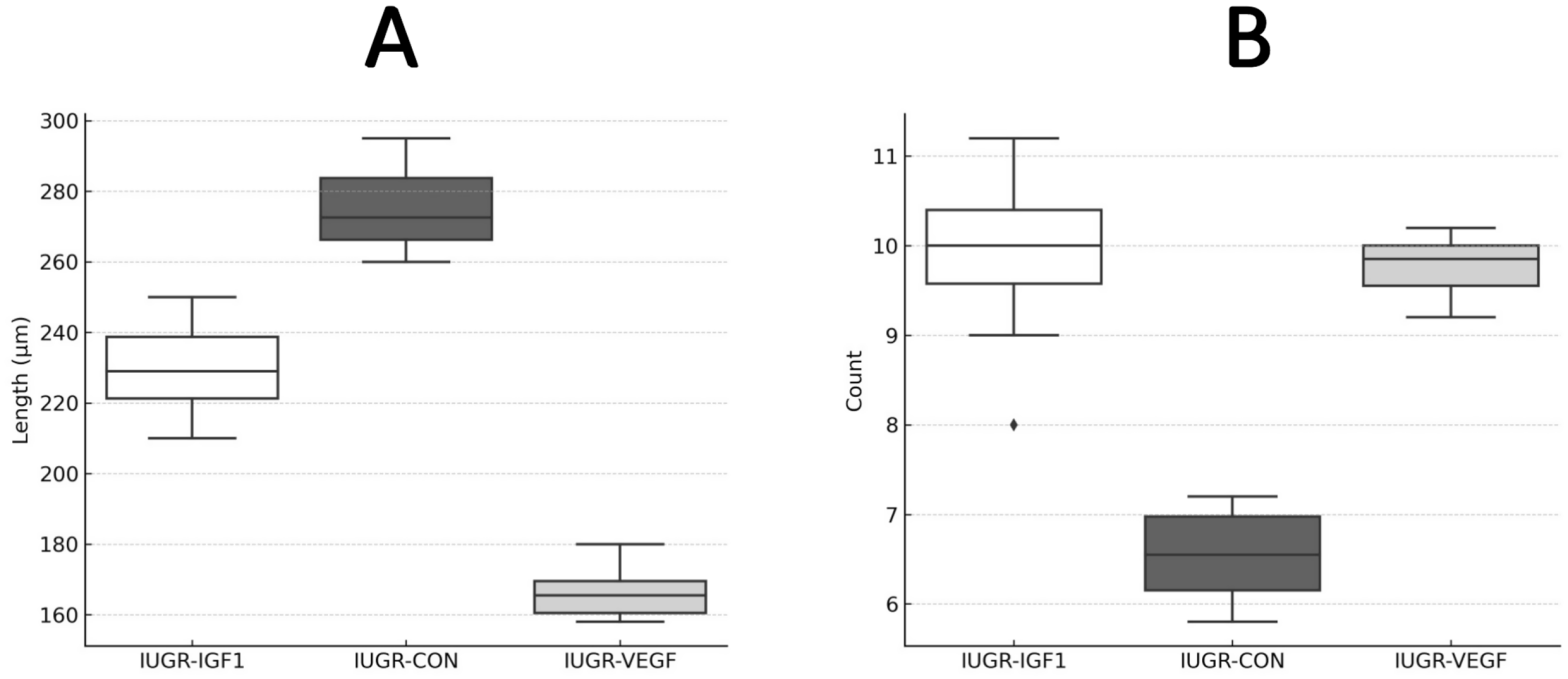
Histomorphometric analysis of intestinal villi at the end of the study in rabbits after receiving IUGR condition (IUGR-CON) and Ad-VEGF (IUGR-VEGF) or Ad-IGF1 (IUGR-IGF1). **(A)** Villus length (μm). **(B)** Number of villi/mm (unit/mm^2^).

Finally, histological assessment of organ development after the intraplacental therapies showed no effects to the liver and kidney but significant differences in the lung and intestine. In the lung (Figure 5), the percentage of parenchyma relative to the alveolar space was similar in all groups, ruling out pathological alterations; however, the treatment in Group IUGR-VEGF increased the number of alveoli/mm^2^ when compared to the other IUGR groups (316.57 ± 145.43 vs. 251.78 ± 79.14 in IUGR-IGF and 224.56 ± 42.90 in IUGR-CON; *p* = 0.055). In the intestine (Figure 6), the treatment in the IUGR-VEGF and IUGR-IGF groups showed a trend to increase both the number of crypts/mm^2^ (*p* = 0.183) and the number of villi/mm^2^ when compared with the IUGR-CON group (*p* = 0.089); however, both the IUGR-VEGF and IUGR-IGF groups showed a significantly shorter villous length and a trend toward a lower crypt depth than in the IUGR-CON group (*p* = 0.045 and *p* = 0.147, respectively).

## 4 Discussion

The results of the present study support the feasibility, safety, and efficiency of the ultrasound-guided administration of adenovirus-mediated gene therapies in a preclinical rabbit model. Our first trial confirmed the feasibility and safety, at both short- and long-term, of the ultrasound-guided puncture at either the placenta or the amniotic fluid; no deleterious effects were found when assessing the pre- and postnatal development of the offspring. The placental route was used in the subsequent trials, despite the lack of differences in feasibility and safety, because technicians were more confident when injecting in the placenta than when puncturing the amnios. The assessment of the efficiency of the ultrasound-guided administration of the adenoviral vector, performed in the second trial, showed a lack of tissue damage and a successful passage and expression of the transgene by Ad at the fetal organs. The third trial supports the efficiency of ultrasound-guided Ad-based gene therapies for preventing or alleviating IUGR-related events and sets the basis for further studies in the field. Most studies are focused on pregnancy and the prenatal period (5); this study is one of the few to evaluate long-term effects (up to 135 days postnatal) on multiple systems (growth, metabolism, and tissue histology).

We choose a rabbit model in the present study because it is considered one of the most suitable and robust models for reproductive preclinical research due to its placental physiology, which more closely resembles that of humans compared to other commonly used laboratory animals. Rabbits, unlike rodents, possess a discoid and hemodichorial placenta, mirroring the early gestational phase of the human placenta (27), as opposed to the hemotrichorial structure observed in rodent models (28). Moreover, the larger fetal size of rabbits when compared to rodents facilitates the administration of intrauterine therapies and the real-time monitoring of fetal development with clinical ultrasound equipment. Additionally, their high fertility rate enables a reduction in the number of pregnant females required, thus aligning with the 3Rs principle of animal experimentation—Replacement, Reduction, and Refinement (29). Such advantages are further supported by the docile temperament of this species, which favors the use of non-invasive imaging techniques (30).

The ultrasound-guided transplacental technique used in the present study induced no harm to mother and fetuses and only a minimal injury to pregnancy structures (puncture point at the placenta) when applied. Previous protocols make necessary the exteriorization of the uterus to enable direct intramural administration of therapies (18, 31), so they required one or more laparotomies during gestation, which implies major surgical burden for both dam and fetus. The intraplacental route has demonstrated promising outcomes in various animal models (16, 26); however, it also implies a laparotomy. Ultrasound-guided intragastric injection techniques (24) are feasible only in sheep fetuses older than 55 gestational days, prompting consideration of more invasive alternatives such as X-ray-guided catheterization via the femoral artery to access the uteroplacental circulation.

There were no other short- or long-term consequences on either the offspring or the mother after the use of ultrasound-guided transplacental technique. Occurrence of long-term effects on fetal development has not been assessed in other techniques previously reported [i.e., oral (1, 2), subcutaneous (5), intravenous (6, 10, 32), or intra-amniotic routes (6, 11, 17)]. Concurrently, maternal safety has not been assessed in the different preclinical models of therapeutic interventions intended to mitigate fetal programming [i.e., sheep (11, 18, 19), guinea pigs (20), rats (1), or rabbits (7, 22)].

The use of our ultrasound-guided technique for the administration of adenoviral vectors was found to be successful, with passage and expression of the transgene at the fetal organs and significant positive effects on organ architecture, post-natal growth, and metabolic traits of IUGR offspring. These effects were found to be more robust when using Ad-VEGF than Ad-IGF-1, which may support these treatments based on improving angiogenesis; the fetal or intraplacental administration of VEGF specifically represents a promising strategy for preventing or counteracting IUGR effects. However, we should highlight that the present trial is only a preliminary study and the results obtained are only the basis for further research on different therapeutic agents and treatment protocols, bearing in mind that only a single administration was used.

In any case, it is noteworthy that the administration of Ad-βGal showed the highest expression of the transgene in the fetal intestine and lungs, followed by the kidneys, liver, heart, and placenta. We can hypothesize that fetal intestine and lungs are particularly exposed to Ad-based therapies because of their relatively low vascularization during intrauterine life (32); a decreased perfusion in these regions likely facilitates vector retention and accumulation, thereby enhancing transgene expression. Blood in the fetal circulatory system is preferentially directed through the *ductus arteriosus*, bypassing the pulmonary circuit; hence, only a small fraction of total blood volume (~6% of total volume) reaches the lungs. Similarly, fetal intestinal circulation receives a reduced blood flow (~7% of total volume) due to the minimal digestive activity during gestation.

Afterwards, the assessment of postnatal features in the offspring showed that, even in the case of a single Ad administration, like in the present study, the VEGF-based therapy evaluated in this study was effective in mitigating IUGR-related impairments. At a systemic level, the therapy administration mitigated the effects of undernutrition on fetal growth; i.e., the reduction in body weight of offspring which were found in the IUGR group of our study as in previous studies (33). At a local level, the effects of therapy administration were found particularly within the lung and intestinal tissues, which were also the tissues with higher expression in our second trial. Our findings are especially important because neonatal health critically depends on the proper maturation of both the respiratory and gastrointestinal systems, which are essential for adequate breathing and oxygenation, postnatal nutrient absorption, and immune defense and are compromised in IUGR offspring. In this way, early intestinal development is vital for optimal gastrointestinal functionality; current research has established a connection between IUGR and reduced nutrient digestibility, as well as increased microbial fermentation in the intestine, in pig models (34, 35). In such models, a 15–20% reduction in the number and length of intestinal villi found in IUGR, together with alterations in the balance between mitosis and apoptosis within the intestinal crypts and villi, have been found to affect nutrient absorption and immune protection in piglets (36, 37). It is noteworthy that, in our present study, the offspring in the IUGR-IGF and IUGR-VEGF groups showed intestinal remodeling with increased villi number; we have to note that the concomitant decrease in villous length has been previously described in previous studies on the physiological dynamics between epithelial turnover and intestinal villous length (38). Overall, our results suggest that the proposed therapies may exert structural remodeling effects in intestinal and pulmonary tissues, potentially contributing to improved functional outcomes in IUGR-compromised fetuses. Hence, these findings pave the way for future studies that include digestibility assays, which may provide valuable insights into intestinal functional development and nutrient absorption capacity and provide improvement in the effects of fetal inflammatory response syndrome (39).

The assessment of postnatal metabolic features in the present study, as in the histological study, were significantly weakened by the lack of plasma and tissue samples in the control groups due to animal welfare limitations imposed by our Ethical Committee, which estimated that our research group had enough comparative data on normal birth-weight, IUGR, and supplemented offspring from previous studies (22, 34, 40, 41). Hence, the present study was based on possible improvement of the IUGR condition by the proposed therapies. In fact, our results clearly showed that the administration of VEGF during intrauterine life demonstrated a protective effect against IUGR-related alterations—again, greater than in the case of IGF-based therapy. Blood HDL concentrations were higher while LDL-c, triglycerides, lactate, and urea were lower after both treatments, but VEGF-based therapy resulted in a more pronounced reduction in NEFA levels. Free fatty acids, or NEFAs, are protein-bound lipid biomolecules that represent approximately 98% of total serum fatty acids and constitute the principal building blocks of triglycerides. The serum fatty acid profile is widely recognized as a biomarker for assessing the risk of various metabolic and cardiovascular disorders (42). NEFA release is typically stimulated by lipoactive hormones, including epinephrine, norepinephrine, glucagon, thyrotropin, and adrenocorticotropic. In this regard, future research could benefit from quantifying the circulating levels of these hormones to elucidate possible variations in endocrine responses associated with VEGF or IGF-1 treatment, thereby contributing to a more comprehensive understanding of their metabolic effects.

## 5 Conclusion

Our present study indicates the feasibility and safety of the ultrasound-guided administration of adenovirus-mediated gene therapies and will aid further investigations involving multiple and/or higher doses in order to achieve significant therapeutic outcomes that could be the basis for translational research into clinical practice.

## Data Availability

The original contributions presented in the study are included in the article/supplementary material, further inquiries can be directed to the corresponding author.
